# MXene/AgNW composite material for selective and efficient removal of radioactive cesium and iodine from water

**DOI:** 10.1038/s41598-023-47075-y

**Published:** 2023-11-11

**Authors:** Sajid Mushtaq, Syed M. Husnain, Syed Asad Raza Kazmi, Yawar Abbas, Jongho Jeon, Jung Young Kim, Faisal Shahzad

**Affiliations:** 1 Division of RI-Applied Research, Korea Institute of Radiological and Medical Sciences (KIRAMS), Seoul, 01812 Korea; 2 Department of Nuclear Engineering, Pakistan Institute of Engineering and Applied Sciences, P. O. Nilore, Islamabad, 45650 Pakistan; 3 Chemistry Division, Directorate of Science, Pakistan Institute of Nuclear Science and Technology (PINSTECH), Islamabad, 45650 Pakistan; 4 Department of Metallurgy and Materials Engineering, Pakistan Institute of Engineering and Applied Sciences, P.O. Nilore, Islamabad, 45650 Pakistan; 5 Department of Physics, Khalifa University, 127788 Abu Dhabi, United Arab Emirates; 6 Department of Chemistry, Kyungpook National University, Daegu 80, Republic of Korea; 7 Research and Innovation Center for Graphene and 2D Materials (RIC2D), Khalifa University, 127788, Abu Dhabi, United Arab Emirates

**Keywords:** Environmental impact, Two-dimensional materials

## Abstract

Toxic fission products, such as cesium (^137^Cs) and iodine (^129^I) are of great concern because of their long half-lives and high solubility in water. The simultaneous removal of Cs and I using a single adsorbent is an area of increasing interest. In this study, MXene/silver nanowire (AgNW) composite was synthesized through physical mixing and employed for simultaneous removal of iodide (I^−^) and cesium (Cs^+^) ions from contaminated water. The MXene/AgNW composite demonstrated excellent adsorption capacities of 84.70 and 26.22 mg/g for I^−^ and Cs^+^, respectively. The experimental data supported the hypothesis of multilayer adsorption of Cs^+^ owing to the inter-lamellar structures and the presence of heterogeneous adsorption sites in MXene. The interaction between I^−^ and the AgNW involved chemisorption followed by monolayer adsorption. MXene/AgNW composite material exhibited promising results in the presence of competitive ions under extreme pH conditions. Thus, synthesized composite materials holds promising potential as an adsorbent for the remediation of radioactive liquid waste.

## Introduction

Fossil fuels, including coal, methane, and petroleum, are essential sources of energy that have contributed considerably to the progress of the human race, as well as to social and economic development. However, owing to continuous industrial development and population growth, conventional energy sources are no longer capable of satisfying the increasing energy demand^1^. Among the various modern energy resources, nuclear energy stands out as one of the best alternatives, owing to its safety, reliability, efficiency, and cost-effectiveness. Although the benefits of nuclear energy are widely acknowledged, the release of hazardous radioactive isotopes resulting from ongoing plant operations and nuclear accidents remains a major environmental concern^2^. Several historic power plant accidents, such as those of, Fukushima in Japan (2011), Chernobyl in Ukraine (1986), and Three Mile Island in Pennsylvania (1979), have had severe impacts on the health of plant workers, nearby residents, and the environment^3–5^. Large concentrations of toxic fission products, such as radioactive cesium (^137^Cs, T_1⁄2_ = 30.17 years) and iodine (^129^I, T_1⁄2_ = 15.7 million years), are released into the environment. The radioactive I^−^ and Cs^+^ are highly soluble in water and can contaminate the food chain through potable water^6^. These radioisotopes have the potential to accumulate in the human body and cause various types of cancers, including leukemia, and mental and metabolic disorders^7^.

To mitigate the presence of these radioisotopes, various technologies have been developed to efficiently remove I^−^ and Cs^+^ ions from aqueous media, including solvent extraction, membrane separation, chemical precipitation, adsorption, and ion exchange^8,9^. However, these methods have limitations. For instance, solvent extraction and chemical precipitation have technical drawbacks, such as low selectivity, poor efficiency, and the production of secondary waste^10^; membrane technology is hindered by high costs^11^. The adsorption technology for the removal of radionuclides is highly appreciated owing to its reported advantages, including easy production, convenient operation, high selectivity, and reasonable cost^12^. Various inorganic, organic and inorganic–organic materials, such as metal–organic frameworks (MOFs), zeolites, and activated carbon have been extensively explored for their ability to remove I^−^ or Cs^+^ ions. However, these adsorbent materials have limitations, such as slow dynamics and, poor adsorption capacity, selectivity, and efficiency^13–15^. For instance, highly porous MOFs have been used to remove I^−^ and Cs^+^; however, their limited solubility in aqueous media, complex production processes, and poor selectivity for competitive ions have hindered their commercial˗scale utilization^16^. Researchers also explored modern materials including metal oxides and hydroxides, molybdenum disulfide, boron nitride nano sheets, and 2D graphene nanomaterials. Nevertheless, the hydrophobic natures of molybdenum disulfide, poor stability of metal oxides and hydroxides, and poor dispersion of graphene in aqueous media have prevented their application in radionuclide adsorbents^17–19^. Consequently, the search for efficient adsorbents for the selective removal of radioactive I^−^ and Cs^+^ is ongoing.

Recently, a vast array of newly introduced two˗dimensional (2D) materials known as MXenes has shown great potential as adsorbents for the removal of radioisotopes. MXenes are represented by the chemical formula M_n+1_X_n_T_x_ (n = 1, 2, or 3), where M represents a transition metal (such as Cr, Zr, V, Mo, and Ti), X represents nitrogen or carbon, and T_x_ denotes surface functional groups, such as fluorine, oxygen, or hydroxyl^20^.MXenes and their composite materials have several beneficial characteristics, including a 2D-layered structure, high specific surface areas, remarkable chemistry, and an abundance of -F, -OH, and -O groups on their surfaces, which provide a good number of active sites^21^. These properties have led to extensive exploration of the application of MXenes and their composite materials in the field of decontamination; the materials have shown promising results in the removal of radionuclides and heavy metals, including ^137^Cs, ^90^Sr, ^152–154^Eu, ^105–107^Pd, ^133–140^Ba, ^232^Th, and ^235–238^U, as well as nonradioactive ions, such as Cr^4+^, Pb^2+^, Cu^2+^, and Hg^2+^, from aqueous media^22–24^. As an adsorbent, MXenes exhibit high chemical, thermal, and radiation stabilities. Ion removal occurs through various mechanisms, such as weak van der Waals interactions, surface coordination, and ion exchange^25,26^. Owing to their extraordinary surface properties, MXene composite materials are anticipated to possess excellent capacity for the simultaneous removal of various toxic radioisotopes. For instance, Rethinasabapathy, et al. developed a composite material based on amino-functionalized polyhedral oligomeric silsesquioxane nanocage and MXene nanosheets for the efficient removal of cesium and strontium ions^27^.

Over the course of several decades, silver and its nanomaterials derivatives have found widespread and significant utility within various domains of engineering and medical sciences^28,29^. The synthesis of silver nanomaterials encompasses an array of structures such as silver nanoparticles^30^, silver nanorods^31^, silver nanocubes^32^, silver nanocoils^33^, and silver nanowires (AgNW)^34^. Among these nanospecies, AgNW has become the central focus of considerable scientific interest due to their distinctive one-dimensional (1D) structure, exceptional mechanical, thermal and optoelectronic attributes, and elevated aspect ratio relative to comparable nanostructures^35^. Derived from the concept of quantum wires, the characterization of AgNW is traditionally rooted in their surface morphology, dimensions, and geometrical properties. Typically, the recognized diameter of AgNW ranges from 10 to 200 nm, with length typically spanning from 10 to 100 μm. AgNW predominantly comprise silver atoms organized in the form of either single or multi twinned crystals, contingent upon the specific manufacturing process employed^36^. These AgNW have demonstrated significant applicability across a wide spectrum of scientific and engineering domains, including the development of advanced sensors, heating devices, electromagnetic shielding systems, and applications within the environmental sciences^37^. In our previous study, we successfully demonstrated the proficient removal of I^−^ using adsorbents containing silver nanomaterials^38^. Based on our previous study, we expect that the synthesized composite material, which combines one-dimensional (1D) AgNW with 2D MXene nanosheets, will exhibit an increased capacity for the simultaneous removal of both I^−^ and Cs^+^ from aqueous media. This synergistic combination is anticipated to outdo the individual efficiencies of MXene or AgNW alone. In this study, we evaluated the capability of MXene/AgNW composite material to concurrently remove I^−^ and Cs^+^ under various physicochemical conditions.

## Results and discussion

### Characterization

**Figure 1:**
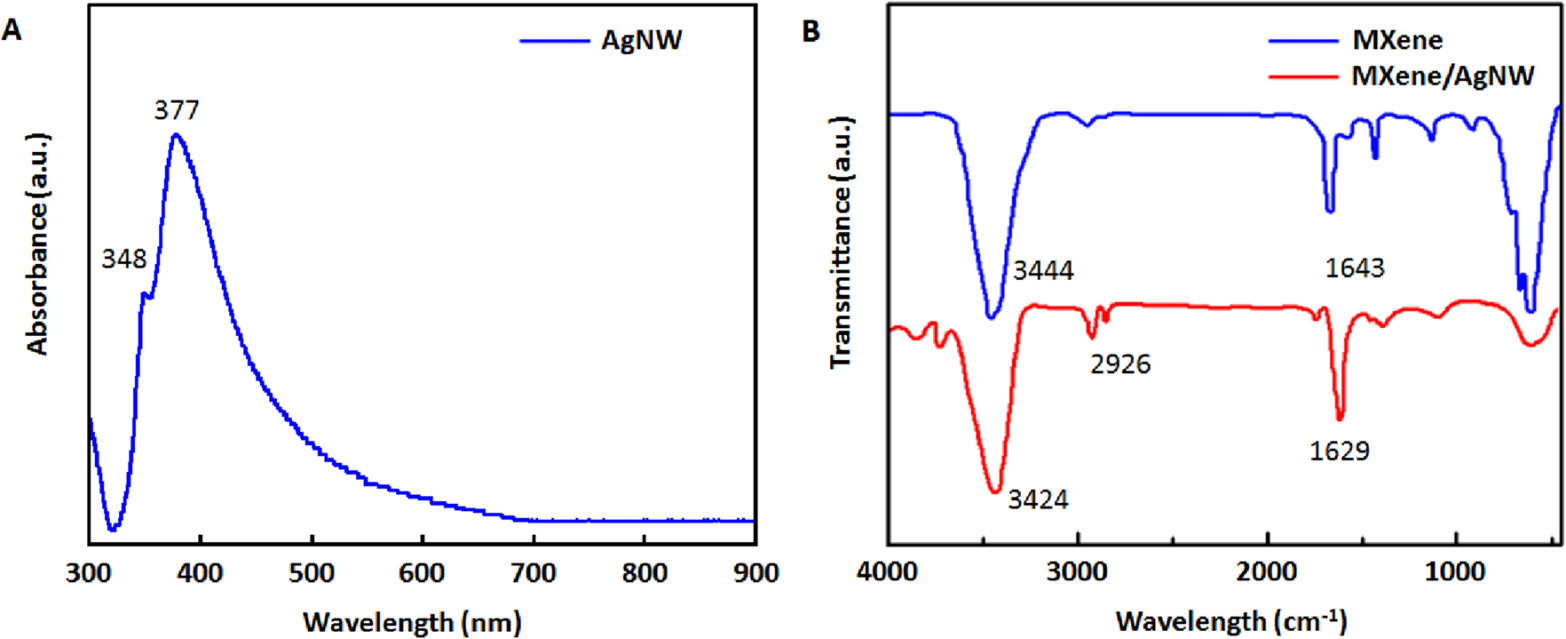
UV–Vis and FTIR spectra of nanomaterials. (**A**) UV–Vis spectrum of AgNW. (**B**) FTIR spectra of MXene and MXene/AgNW composite material.

The MXene/AgNW composite was synthesized using MXene and AgNW. The UV–Vis spectrum of the freshly prepared AgNW displayed a prominent peak at 377 nm and smaller shoulder at 348 nm (Fig. 1A), both of which corresponded to surface plasmon absorption linked to the short axis of the AgNW^39^. FTIR spectroscopy was used to examine the surface modification of the MXene and MXene/AgNW composite. MXene exhibited a characteristic peak (3444 cm^−1^) in the IR spectrum, which was ascribed to the hydroxyl (–OH) group. Another peak (1643 cm^−1^) confirmed the presence of a hydroxyl group and hydrogen bonding with water molecules. The 1150 cm^−1^, 701 cm^−1^, and 636 cm^−1^ peaks were attributed to Ti–F, Ti–O, and Ti–C, respectively^40^ (Fig. 1B). For the MXene/AgNW composite material, a drastic decrease was observed in the intensity of the hydroxyl group vibration peaks (3424 and 1629 cm^−1^ respectively). This decrease confirms the formation of hydrogen bond between MXene and the AgNW.

**Figure 2:**
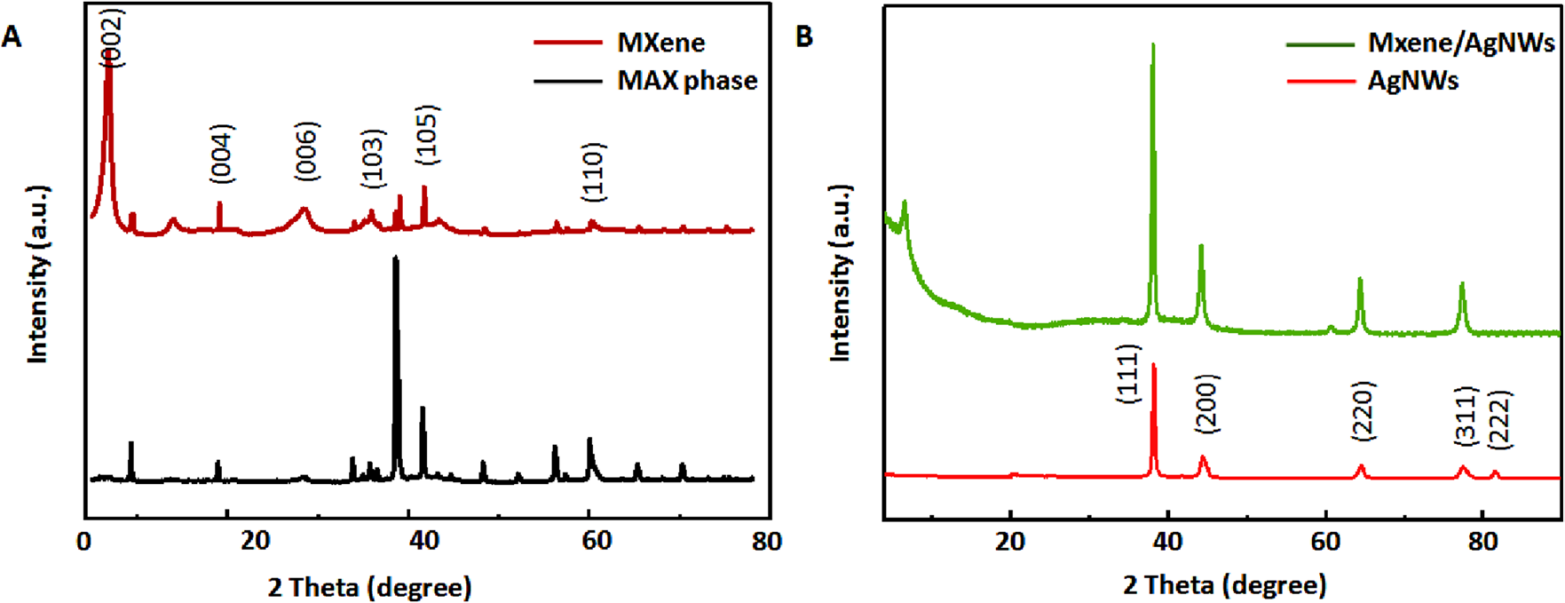
X-ray diffraction (XRD) spectra of nanomaterials. (**A**) XRD spectrum of Ti_3_C_2_T_x_ (MXene). (**B**) XRD spectra of AgNW and the MXene/AgNW composite material.

Figure 2A shows the XRD patterns of MAX and MXene. MAX (Ti_3_AlC_2_) showed characteristics peaks at a 2θ value of 76.61° (116), 71.55° (109), 60.53° (110), 52.97° (106), 44.17° (104), 38.52° (103), 33.80° (100), 19.08° (004), and 9.95° (002). The majority of these peaks disappeared in the XRD pattern of MXene, suggesting successful etching with a downward shift in the (002) peak, confirming an increase in the interlayer spacing due to the removal of Al metal. The overall XRD spectrum of Ti_3_C_2_T_x_ MXenes was in good agreement with previously published XRD data^41^. The AgNW XRD pattern was consistent with previous reports^42^, where the characteristic peaks appeared at 2θ = 38.19°, 44.39°, 64.61°, 77.51°, and 81.51°, corresponding to the crystal planes indexed as (111), (200), (220), (311), and (222), respectively. The XRD spectrum of the MXene/AgNW composite material showed footprints for both MXene and AgNW (Fig. 2B).

**Figure 3:**
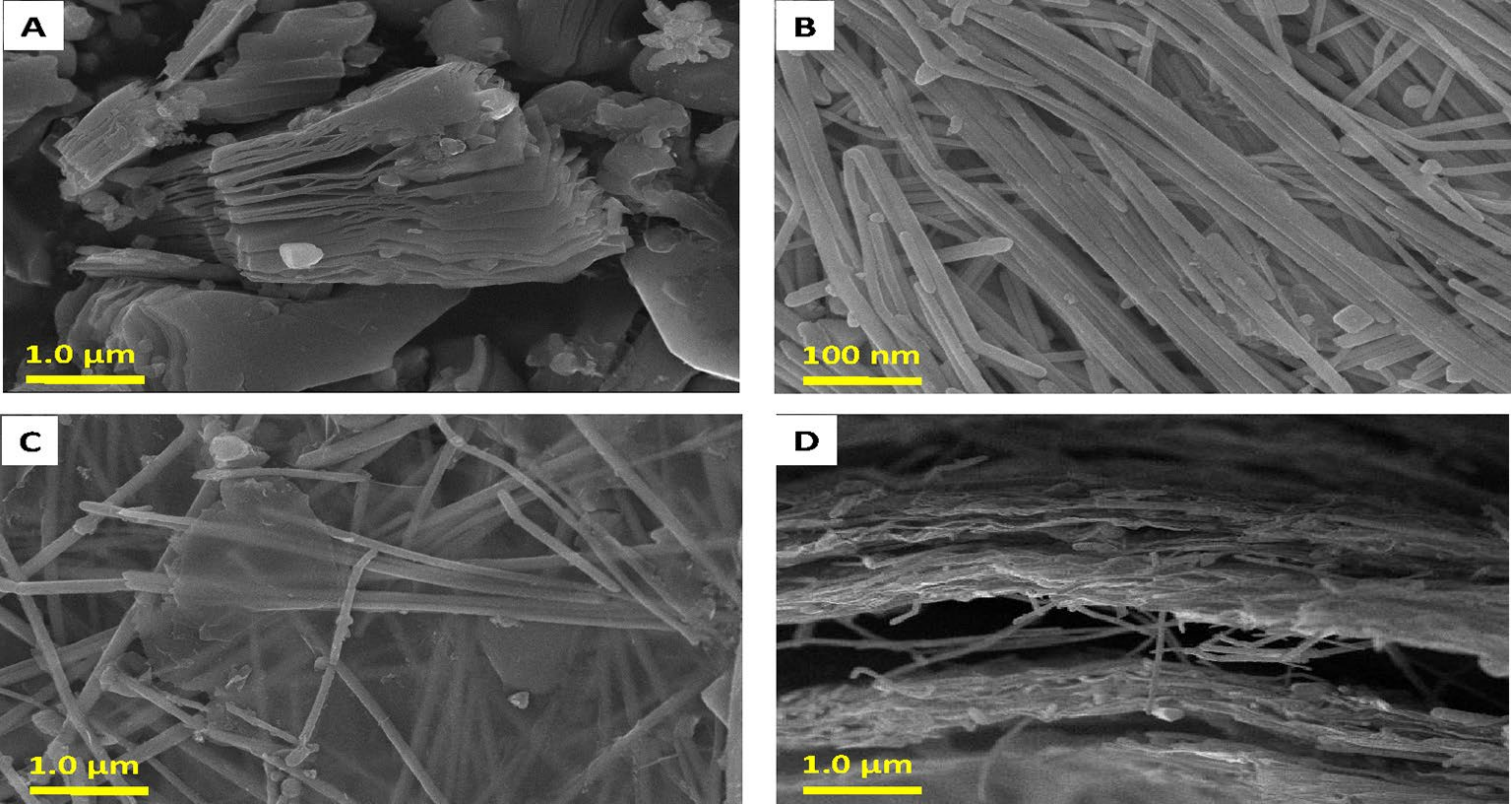
High-resolution scanning electron microscopy images of (**A**) MXene, (**B**) AgNW, and (**C**,**D**) MXene/AgNW composite material.

High-resolution SEM images showed the morphologies of the samples (Fig. 3). Upon etching, the pure MXene was observed to exhibit a smooth 2D layer with a lateral size of 1–2 μm (Fig. 3A). The surface of the AgNW was 1D, smooth, and did not contain impurities. Detailed EDS and elemental mapping analysis of AgNW and MXene provided the elemental composition and spatial distribution of the elements (Figs. S1 and S2) and confirmed the successful synthesis of the nanomaterials without impurities. Finally, the AgNW was observed to be evenly distributed on the MXene sheet (Fig. 3C,D). The presence of ample functional groups in MXene was responsible for the uniform deposition of AgNW, allowing effective coordination through hydrogen bonding. EDS was performed on the MXene/AgNW to determine the elemental content and distribution of various elements on the surface. Al was not found in the MXene, suggesting successful etching and washing. The oxygen content partly resulted from the self-oxidation of MXene during synthesis and partly from the AgNW surface chemistry. The presence of Ag indicates that the AgNW was successfully immobilized on MXene. Elemental mapping and distribution analyses validated the presence of Ag, C, F, O, and Ti (Fig. S3). The Ag, O, and Ti mappings resembled and completely overlapped with the SEM images. EDS analysis confirmed the smooth dispersion of AgNW on the MXene sheets through hydrogen bonding. The presence of F was due to the surface termination originating during the chemical etching process.

**Figure 4:**
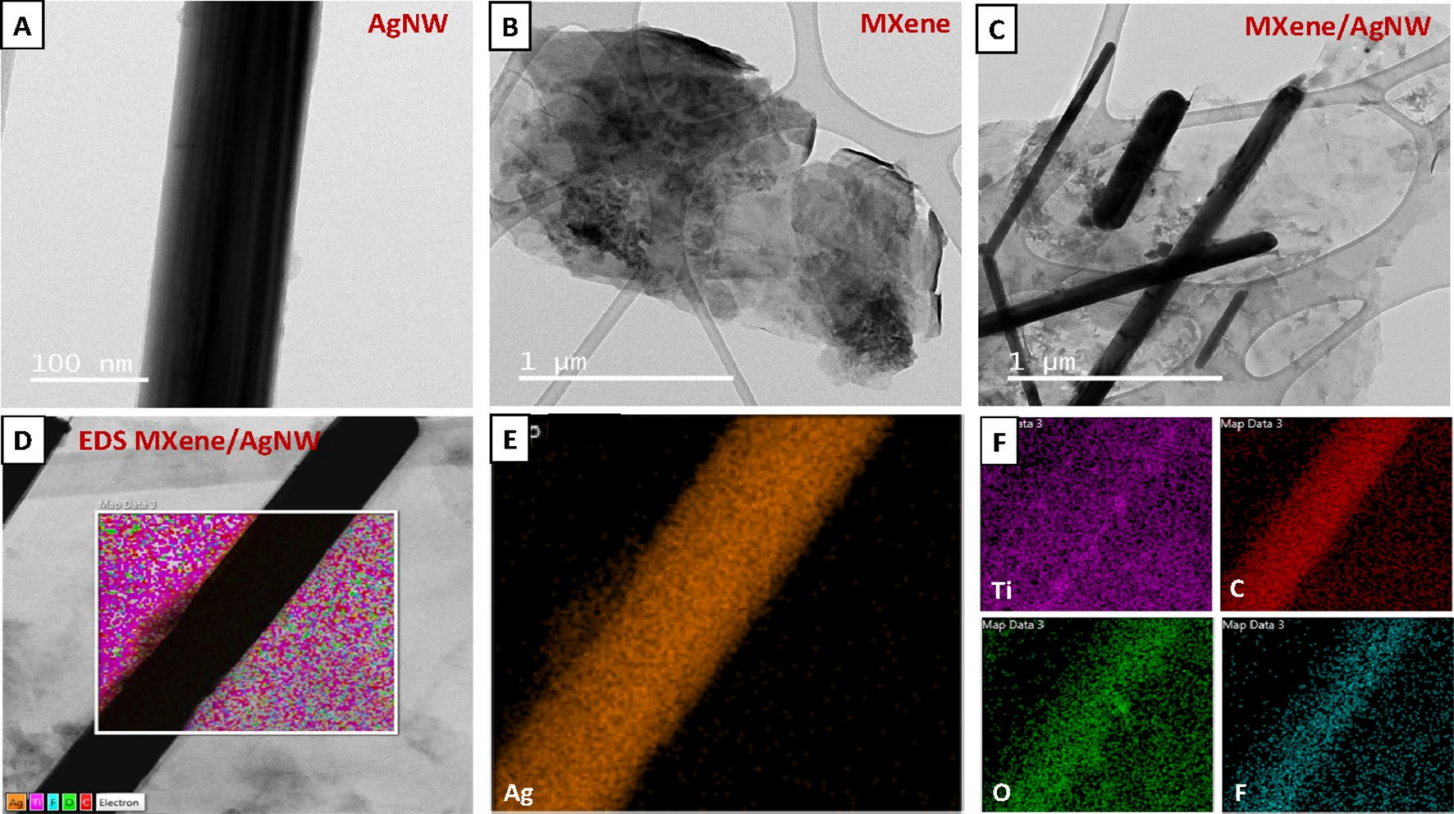
TEM images of (**A**) AgNW, (**B**) MXene, (**C**,**D**) MXene/AgNW composite, and (**E**,**F**) elemental mapping for MXene/AgNW composite, showing the presence of Ag, and Ti, C, O, and F, respectively.

We conducted a TEM analysis to study the structural characteristics of the prepared materials (Fig. 4). The TEM images of the AgNW confirmed their smooth, wire-like 1D structure. The growth mechanism of the AgNW began with the reduction of Ag^+^ to Ag in the presence of ethylene glycol. The adsorption of PVP onto Ag facilitated the formation of cyclic penta-twinned-crystal seeds. These nano-crystals play a crucial role in controlling the diameter of the nanowires and promoting longitudinal growth^43^. The center of a single nanowire appeared much thicker and darker than its side walls (Fig. 4A), thus supporting the mechanism of formation. Under TEM, pure MXene displayed a sheet-like shape after etching, indicating successful etching (Fig. 4B). In MXene/AgNW both MXene sheets and AgNW can be distinguished (Fig. 4C). Numerous AgNW were evident on the surface of the layered material. Elemental mapping of the MXene/AgNW composite showed a strong signal for Ag in a magnified image of the dark, thus confirming the presence of AgNW. Ti, the main element in MXene had exhibited the highest content (Fig. 4D–F).

### Adsorption behavior of the MXene/AgNW composite material

#### General adsorption study

**Figure 5:**
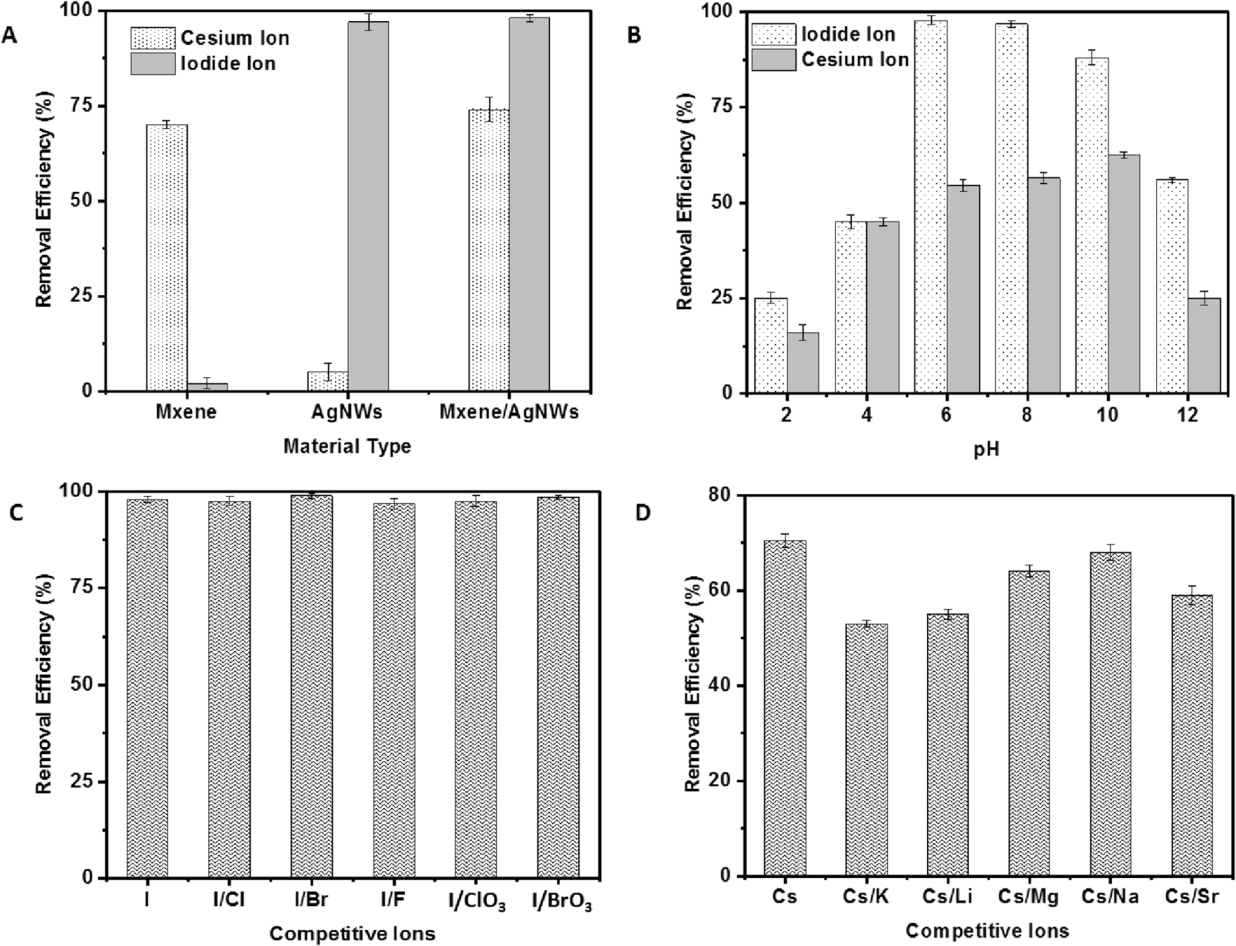
The removal of Cs^+^ and I^−^ under (**A**) the use of various materials (n = 3) and (**B**) various pH levels using the MXene/AgNW composite (n = 5). (**C**) The removal efficiency of I^−^ (n = 3) and (**D**) Cs^+^ (n = 3) in the presence of competitive ions using the MXene/AgNW composite.

We investigated the adsorption of Cs^+^ and I^−^ ions at neutral pH and room temperature, with removal efficiency measured over a range of 5–250 min for MXene, AgNW, or MXene/AgNW composite. The removal efficiency of Cs^+^ by MXene or MXene/AgNW composite was detected within minutes of interaction, resulting in removal efficiencies of 63% and 61%, respectively. This excellent removal efficiency was attributed to the hydrophilic nature, large surface area, and the presence of functional groups on the surface of MXene, which could host an abundance of Cs^+^ (Fig. 5A). The SEM, EDS, and elemental mapping analyses showed that the structural morphology and elemental content of the adsorbent remained unaltered after the experiment. However, a distinct signal for Cs^+^ was detected in MXene, indicating effective adsorption of the Cs (Fig. S4). Interestingly, AgNW alone had a negligible effect on Cs^+^ adsorption. Subsequently, we treated the three adsorbents with I^−^ under various experimental conditions and found that MXene removed negligible amounts of I^−^, where, AgNW and MXene/AgNW composite demonstrated excellent removal efficiencies. A prominent absorbance peak was observed at 225 nm corresponding to I^−^, which disappeared completely after treatment with AgNW or the MXene/AgNW composite (Fig. S5). The AgNW composite successfully removed I^−^ within a few minutes with > 99% removal efficiency, and a plateau was observed after 60 min (Fig. 5A). SEM, EDS, and elemental mapping analyses of the AgNW further confirmed the successful removal of I^−^ (Fig. S6). In the final analysis, we found that the MXene/AgNW composite successfully removed both the Cs^+^ and I^−^ with removal efficiency comparable to that of MXene and AgNW. The SEM images revealed that the structural morphology and elemental content of the MXene/AgNW composite remained intact; however, the presence of I and Cs was observed in EDS and elemental mapping after treatment of the sample (Fig. S7).

### Effect of pH

We assessed the impact of pH on the adsorption of Cs^+^ or I^−^ through a series of experiments with pH 2.0–12.0. We treated the adsorbent with 0.05 mg (10 mL) of Cs^+^ or I^−^ for 60 min and found that more than 90% I^−^ was absorbed between pH 6.0 to 8.0, and on the whole remained above 85% at pH 5.0–10.0 (Fig. 5B). Interestingly, beyond pH 10.0, the adsorption decreased significantly, and less than 50% adsorption was observed at pH 12.0. This is because pH values considerably influence the deprotonation/protonation process of MXene/AgNW. Moreover, during adsorption, the attainment of the most stable state occurs when there is an ideal balance of electron energy states between the target ions and adsorbent within the solution^44^. Under extreme basic conditions, the equilibrium state is compromised and consequently a sudden decrease in adsorption occurs. Moreover, the rate and degree of silver oxidation play pivotal roles in the adsorption of I^−^, whereas, the interaction of I^−^ with metallic silver in aqueous media depends upon the oxidizing conditions. Hence, dissolved oxygen as well as the pH of the mixture influences the oxidation process^45^. It can be seen that as the solution pH increases above 9.0, a gradual reduction in oxidation becomes evident, where high concentration of hydroxyl ions act as scavengers, hindering the oxidation process. This process results in the gradual degradation of AgNW at pH > 9.0, which dissolves Ag and does not remove I^−46^. Similarly, high dissolution of Ag was observed at pH < 4.

Similarly, a significant influence of pH was observed on Cs^+^ adsorption. This is because considerable change in pH can affect the charge densities of the functional groups on the MXene surface. The adsorption of Cs^+^ below 15% was observed under acidic conditions (Fig. 5B). Under acidic pH, the surface of the adsorbent could be covered by hydrogen ions, which limits the adsorption of Cs^+^ due to repulsive forces^47^. Interestingly, with an increasing pH (up to 10) the adsorption of Cs^+^ increased. However, a sharp decrease was observed beyond pH 11.0, which was likely due to the instability and high dissolution of the MXene/AgNW. Moreover, the significant decrease in the adsorption of Cs^+^ under extremely basic conditions may be attributed to the development of cesium hydroxide (Cs(OH)_2_^−^), which has no affinity for the adsorbent and limits Cs^+^ adsorption owing to electrostatic repulsion^48^.

### Effect of competitive ions

We explored the impact of coexisting ions on the removal of Cs^+^ and I^−^. Iodide removal was not considerably affected by any of the competing anions at the same concentration (Fig. 5C). Although the I^−^ removal kinetic was slower in the initial stages of adsorption for chlorate anions, they attained a maximum adsorption capacity equivalent to that of the other ions. To investigate the effects of competing chloride anions, we used an excess concentration compared to the starting concentration of iodide ions (I^−^:Cl^−^ the ratio of approximately 1:1000). Interestingly, excess concentration of competing chloride anions did not impede the removal effectiveness of the adsorbent, likely because the reaction between iodide and silver oxide has a lower Gibbs energy (− 32 kJ/mol) and high affinity for the surface of the material, along with a relatively small radius compared to other competing anions, such as chloride, fluoride, bromide, and bromate ions^49^. The results indicate that MXene/AgNW possesses a high affinity toward I^−^ and selectively precipitates until I^−^ is unavailable. The high selectivity of AgNW towards I^−^ may be attributed to the high solubility of silver iodide (AgI) precipitates compared with that of other competing anions. In either scenario, the precipitation of AgI represents the kinetically favorable reaction that occurs first.

To determine the efficiency and specificity of the MXene/AgNW composite toward Cs^+^, adsorption studies were performed in the presence of the competitive cations Sr^2+^, Mg^2+^, K^+^, Na^+^, and Li^+^, with concentration tenfold that of the Cs^+^. The adsorption of Cs^+^ was higher than that of any competitive ion except K^+^ (Fig. 5D). The adsorption of Cs^+^ decreased in the presence of K^+^ because the hydrated ion radi of K^+^ (0.33 nm) and Cs^+^ (0.32 nm) are almost equivalent^50^. The Brunauer–Emmett–Teller analysis confirmed that the average pore diameter of MXene ranged from 0.736 to 0.739 nm^51^. These results suggest that MXene has the same capacity to accommodate K^+^ and Cs^+^ in its pores and interlayer spacing.

### Adsorbent isotherms and adsorption kinetics

**Figure 6:**
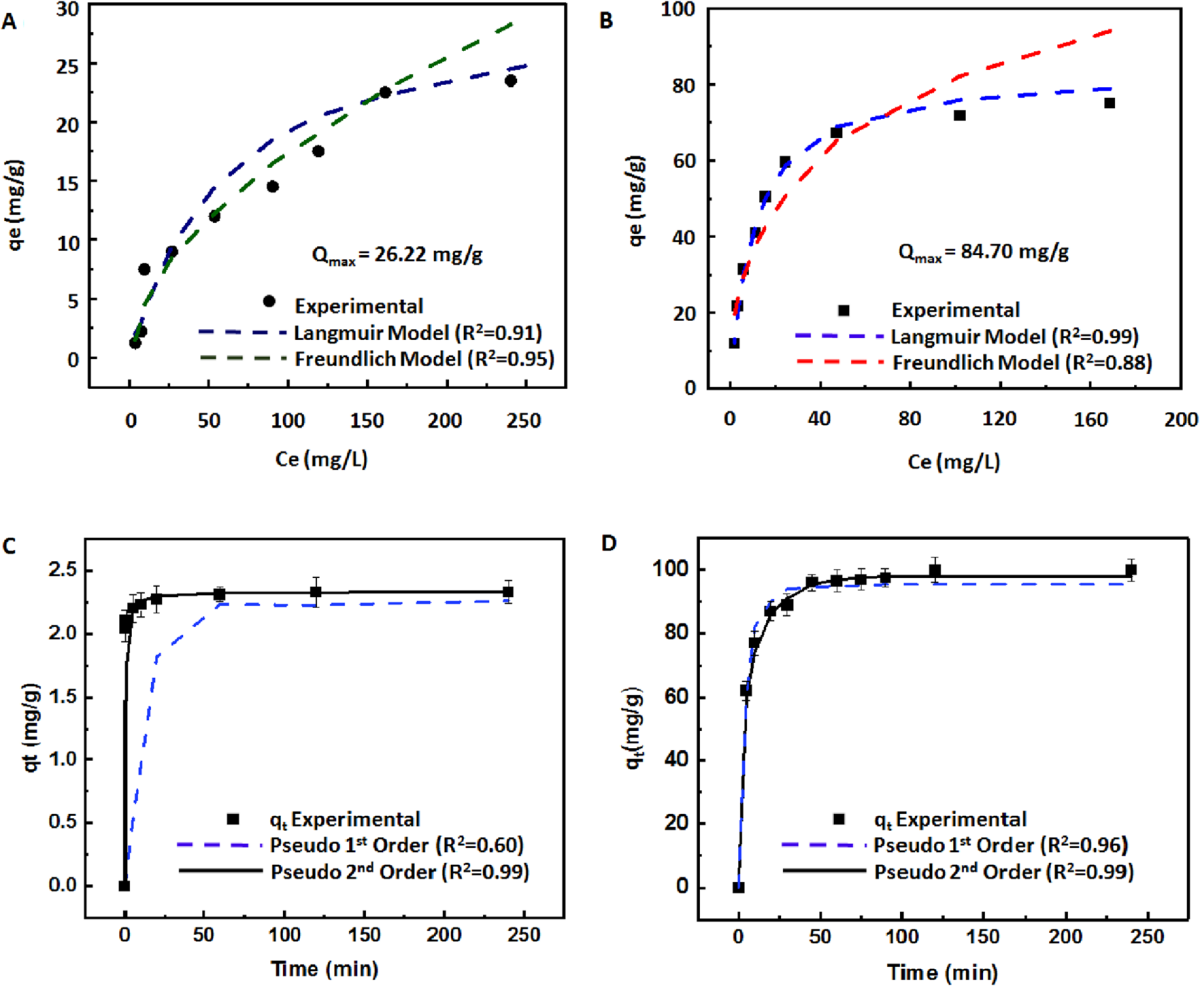
Freundlich and Langmuir isotherms and adsorption kinetics for the MXene/AgNW. Freundlich and Langmuir isotherms for (**A**) Cs^+^, and (**B**) I^−^. Adsorption kinetics of (**C**) Cs^+^, and (**D**) I^−^.

An adsorption study was carried out to investigate the mechanism and maximum adsorption capacity of the MXene/AgNW composite material for Cs^+^ and I^−^. We performed adsorption isotherm experiments, from which we derived correlation coefficients (R^2^) of 0.95 and 0.91 for the Freundlich and Langmuir isotherm models of Cs^+^, respectively (Table 1). The experimental data for Cs^+^ ion adsorption exhibited a superior fit with the Freundlich model (Fig. 6A), indicating that the adsorption behavior of the MXene/AgNW composite material is heterogeneous and multi-layer adsorption is possible. The presence of a variety of functional groups, including oxygen, fluorine, and hydroxyl groups, with different binding energies and chemistries, contributes to this heterogeneous adsorption behavior. We anticipated that the minimum binding energy sites would be occupied first during adsorption and that adsorption would decrease exponentially until the maximum adsorption capacity (26.22 mg/g) of Cs^+^ ion was reached. Moreover, the Freundlich constant for Cs^+^ was greater than 1 (n = 1.8), indicating the favorable adsorption of Cs^+^ onto the composite material. The remarkable adsorption behavior of Cs^+^ on MXene arises from a combination of various mechanisms, encompassing ion-exchange, electrostatic interactions, and complex formation^52,53^. This is also attributed to the 2D structure of MXene, which provides unique inter-lamellar spaces that can trap Cs^+^^54^. Furthermore, the presence of -F, -OH, and -O moieties in the form of Ti_n+1_C_n_T_x_ (n = 1, 2, or 3), (T = F, O, or OH), which was confirmed by FTIR analysis, suggests the involvement of ion-exchange mechanisms in cesium ion adsorption^55,56^. Similar trials were conducted to explore the adsorption behavior of I^−^ ions on the MXene/AgNW composite. The Langmuir isotherm model provided an excellent fit to the experimental data over the entire concentration range (R^2^ = 0.99). The *Q*_*max*_ of the composite material for I^−^ was 84.70 mg/g based on the Langmuir model, which is consistent with the adsorption kinetics results (Fig. 6B). The rapid initial removal of iodide ions was ascribed to its fast reaction with Ag on the surface of the AgNW; however, the constrained ion diffusion towards the inner surface significantly attenuated the removal rate. Consequently, the initial kinetics was fast, but eventually plateauted. The relatively slow adsorption at a later stage may be attributed to the complex removal mechanism of I^−^. This mechanism encompasses an initial oxidation of AgNW to form a silver oxide, which is succeeded by the dissolution and generation of Ag. Ultimately, it involves a chemical reaction between oxidized Ag and I^−^ to produce AgI^57^. The formation of AgI creates a non-porous layer due to the substantial molar volume of iodine. This layer hinders the subsequent diffusion of oxygen and I^−^ required for ongoing reactions, thereby constraining the overall removal rate^58^. Next, we investigated the adsorption kinetics of MXene/AgNW for Cs^+^ and I^−^ for various contact times (ranging from 5 to 250 min) at 25 °C. The swift removal of Cs^+^ by the MXene/AgNW within the initial contact phase can be attributed to the abundance of vacant adsorbent sites. Equilibrium was achieved in approximately 30 min (Fig. 6C). The kinetics of Cs^+^ adsorption was assessed using PFO and PSO models. The PFO kinetic model exhibited a limited match with the experimental data. However, the PSO kinetic model exhibited the best fit and effectively described Cs^+^ adsorption (R^2^ = 0.99). Notably, the calculated Q_e_ value (2.4 mg/g) closely corresponded to the experimental value (2.45 mg/g). These outcomes suggested that the PSO kinetics predominantly reflect chemisorption as the primary governing factor for the adsorption of Cs^+^ onto the MXene/AgNW composite^59,60^.

**Table 1:** Adsorption isotherm parameters for MXene/AgNW composite material.

Ion	Type of Isotherm	Parameters	R^2^ (coefficient of determination)
Cesium	Langmuir model	Q_max_ = 26.22 mg/g K_L_ = 0.56 L/mg	0.910
	Freundlich model	n = 1.8 K_f_ = 1.41 L/mg	0.951
Iodide	Langmuir model	Q_max_ = 84.5 mg/g K_L_ = 0.91 L/mg	0.995
	Freundlich model	n = 2.6 K_f_ = 148 L/mg	0.871

The MXene/AgNW composite exhibited a notable I^−^ adsorption effect (Fig. 6D), evidenced by an initial rapid adsorption rate within the first 60 min. This accelerated rate can be attributed to the abundance of active sites (Ag_2_O_x_ species) for effective interaction with I^−^. A significant fraction of Ag_2_O_x_ reacted with I^−^ in the solution, forming AgI precipitates. However, the adsorption rate reached a plateau between 60 and 260 min, owing the extensive consumption of the active sites. Evaluation of the PSO model (R^2^ = 0.99) showed a closer alignment with the experimental results. This observation suggested that the removal of I^−^ was predominantly achieved by chemical adsorption.

Overall, MXene/AgNW composite exhibited a substantial and comparable adsorption capacity for Cs^+^ and I^−^, surpassing that of several previously reported adsorbents (Table S1).

### Treatment of radioactive sample

**Figure 7:**
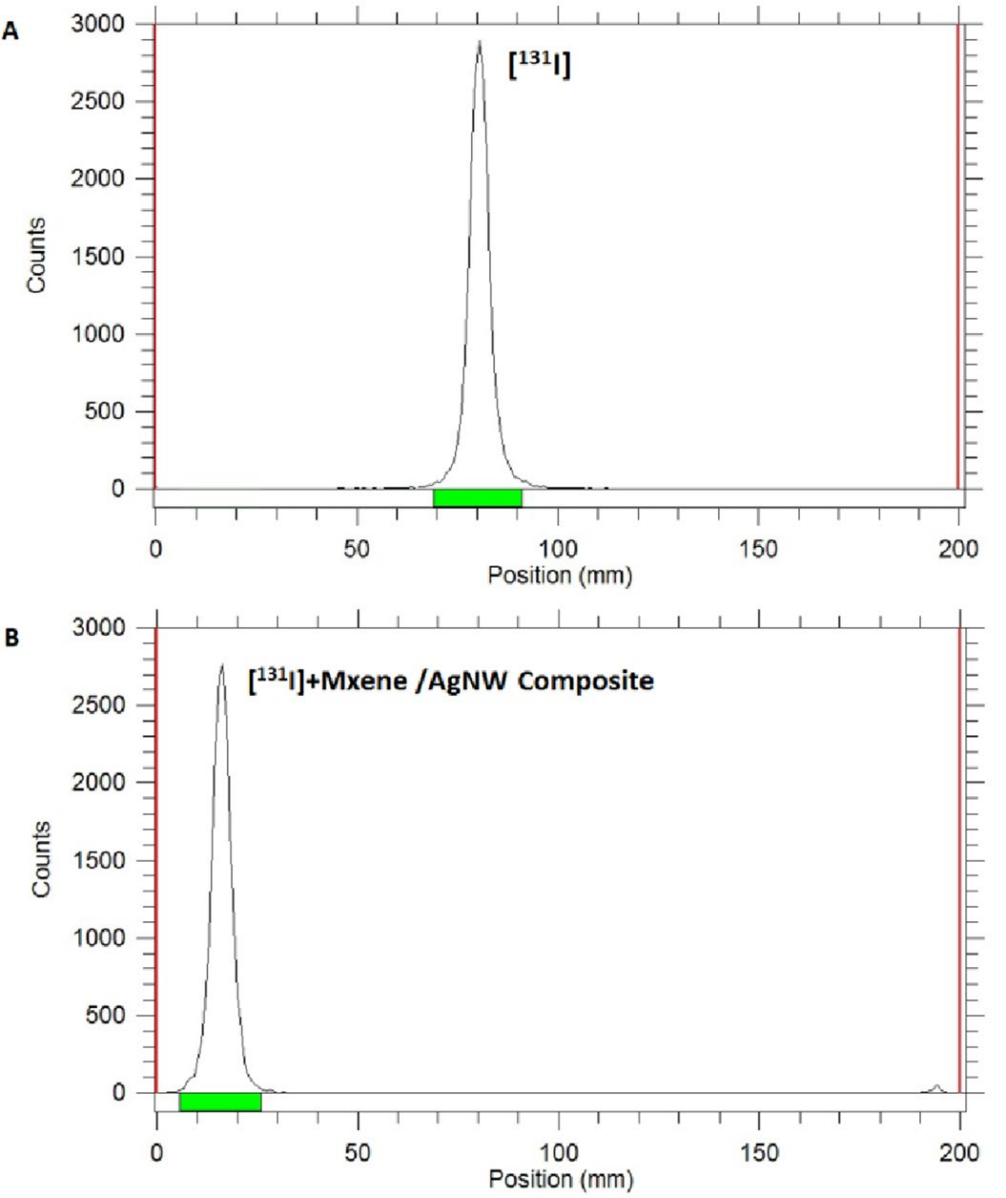
Radio-TLC results for the removal of the radioactive iodide ion (^131^I^−^) using the MXene/AgNW composite (eluent: 0.9% saline) (**A**) Radio-TLC of free ^131^I^−^ (**B**) Radio-TLC of MXene/AgNW composite after the adsorption of ^131^I^−^.

A supplementary series of tests were performed to assess the effectiveness of the MXene/AgNW composite as an adsorbent for radioactive specimens. Because a suitable short-lived Cs radioisotope was unavailable, the trials were exclusively performed using the short-lived radioactive iodine (^131^I, T_1/2_ = 8 days). The aqueous sample containing radioactive iodine exhibited a notable reduction upon interaction with the MXene/AgNW composite, causing the radioactive iodine signal to transition from 80 to 15 mm (Fig. 7). This procedure was replicated 10 times by maintaining consistent levels of radioactivity and using the same adsorbent, resulting in a consistently high removal efficiency of over 95%.

## Materials and methods

### Chemicals and equipment

Titanium aluminum carbide 312 (Ti_3_AlC_2_), formic acid (reagent grade), lithium fluoride (LiF), silver nitrate (AgNO_3_), sodium iodide (NaI), potassium iodide (KI), cesium bromide (CsBr), sodium hydroxide (NaOH, 48%), hydrochloric acid (HCl, 37%), polyvinylpyrrolidone (PVP, 40,000 MW), sodium chloride (NaCl), and ethylene glycol were procured from Sigma-Aldrich USA. ^131^I was purchased from PINSTECH (Pakistan). The specific radioactivity of [^131^I]NaI was 1 mCi/μmol and was supplied as aqueous 0.1 M NaOH. Field Emission Scanning Electron Microscope (FE-SEM Hitachi SU8230) was used to study the microstructure and the morphology of the synthesized nanomaterials. The functional groups on the surface of the adsorbents (MXene and MXene/AgNW) were identified using Fourier transmission-infrared (FT-IR)/ near infrared (NIR) spectrophotometer (PerkinElmer Frontier), with a wavelength range of MIR 4000–400 cm^−1^. PANalytical X’Pert PRO-MPD was used to obtain X-ray diffraction (XRD) data, and Metrohm 780 pH meter was to measure the pH. The concentration of Cs^+^ in the solution was measured using a HITACHI Z-2000, Atomic Absorption Spectrometer. To investigate the structural properties of the nanomaterials, transmission electron microscopy (TEM) using the Tecnai G2 instrument from the Netherlands was employed. Finally, the concentration of I^−^ before and after treatment with the adsorbent was measured using Thermo Fisher Scientific™ Evolution™ 201/220 UV–Visible (UV–Vis) Spectrophotometers, whereas an AR-2000 Radio-TLC imaging scanner was used to measure the radioactivity of iodine [^131^I].

### Synthesis of Ti_3_C_2_T_x_ MXenes

MXene (Ti_3_C_2_T_x_) was synthesized using an in-situ HF method as previously reported^61^. Briefly, MAX and LiF powders were used at a 1:10 molar ratio. First, the LiF powder was dissolved in 9.0 M HCl (25 mL) in a polypropylene bottle and stirred at 40 °C for 15 min. Next step MAX powder was gradually added to the LiF/HCl mixture under constant stirring. The reaction mixture underwent continuous stirring at 40 °C in an oil bath for 24 h. Upon completion of the etching process, the mixture was subsequently washed with deionized water, followed by centrifugation at 6000 rpm for 4–5 cycles until the solution reached a pH of 6. The multi˗layered Ti_3_C_2_T_x_ MXene was separated using a membrane filter (cellulose acetate, pore size: 0.2 µm).

### Synthesis of silver nanowires (AgNW)

High-yield AgNW was synthesized as previously described^62^ using freshly prepared silver chloride (AgCl). Briefly, aqueous AgNO_3_ solution (0.5 M, 5 mL) was treated with aqueous NaCl solution (1.0 M, 5 mL) and continuous mixing was applied at 1000 rpm in the dark for 5 min. The AgCl precipitate was filtered, washed with ultrapure water, and dried under a vacuum. To synthesize of the AgNW, PVP (330 mg) was dissolved in ethylene glycol (25 mL) and the mixture was mixed at 1000 rpm and 170 °C for 10 min. Freshly prepared AgCl (20 mg) was then added to the solution. After 5 min, 110 mg of AgNO_3_ powder was added, and the solution was stirred at 1000 rpm and 170 °C for 25 min. UV–Vis spectrophotometer was used to confirm the formation of AgNW.

### MXene/AgNW composite

An aqueous solution of the freshly prepared AgNW (10 mg, 10 mL) was mixed with an aqueous solution of MXene at various concentrations under continuous stirring (1000 rpm, pH 7.0) at 25 °C for 30 min. The composite material was subjected to repeated washing using ultrapure water and centrifuged at 5000 rpm for 10 min.

### Adsorption of nonradioactive cesium and iodine using various materials

The adsorption of nonradioactive I^−^ or Cs^+^ onto MXene, AgNW or MXene/AgNW composite material was studied using various concentrations of adsorbent, Cs^+^ or I^−^. All experiments were conducted in triplicate at ambient temperature. In a typical experiment, 10-20 mg of adsorbent was treated with a 10 mL solution of the respective ion (5 mg/L) in deionized water at normal temperature and pH. The adsorption efficiency of the MXene/AgNW composite material was evaluated under various conditions, such as pH (2–12), time (5–240 min), and in the presence of competitive ions (10 mg/L of Cl^−^, Br^−^, F^−^,ClO_3_^−^, BrO_3_^−^, Li^+^, Na^+^, K^+^, Mg^2+^, or Sr^2+^). Upon completion of the experiments, the adsorbent was retrieved via centrifugation and filtration. The I^−^ concentration was determined using UV–Vis spectroscopy (λ_max_ = 225 nm), and an Atomic Absorption Spectrometer (AAS) was used for Cs^+^ analysis. The percentage removal efficiency (% RE) and adsorption capacity at equilibrium, Q_e_ (mg/g), were calculated using Eqs. (1) and (2) respectively.

1 $$\mathrm{\% RE }= \frac{{\text{(C}}_{\text{o}}\text{- }{\text{C}}_{\text{e}}\text{)}}{{\text{C}}_{\text{o}}} \times 100$$

2 $${\mathrm{Q}}_{\mathrm{e}} = \frac{{(\mathrm{C}}_{\mathrm{o}}- {\mathrm{C}}_{\mathrm{e}})}{\mathrm{m}}\times \mathrm{ V}$$

Here, *C*_*0*_ (mg/L) is the initial concentration of the target ions, *C*_*e*_ (mg/L) is the final concentration of target ions in the aqueous solution at time *t*, *V* (L) is the volume of the solution, and *m* (g) represents the mass of the adsorbent material.

### Adsorption isotherm studies

For the adsorption isotherm calculation, 5 mg of MXene/AgNW composite material was treated with 100 mL of Cs^+^ or I^−^ solution at various initial concentrations (*C*_*o*_, 100–200 ppm) with a steady increment of 10 ppm. The adsorption of the target ions (*Q*_*e*_) was calculated using Eq. (2). Equilibrium adsorption was calculated using the Freundlich (Eq. 3) and Langmuir (Eq. 4) isotherm models.

3 $${\mathrm{lnQ}}_{\mathrm{e}}=\mathrm{ ln}{\mathrm{K}}_{\mathrm{F}} + \frac{1}{\mathrm{n}}{\mathrm{lnC}}_{\mathrm{e}}$$

4 $$\frac{{\mathrm{C}}_{\mathrm{e}}}{{\mathrm{Q}}_{\mathrm{e}}}= \frac{{\mathrm{C}}_{\mathrm{e}}}{{\mathrm{Q}}_{\mathrm{max}}} + \frac{1}{{\mathrm{Q}}_{\mathrm{max }}{\mathrm{K}}_{\mathrm{L}}}$$

Here, *C*_*0*_ and *C*_*e*_ (mg/L) are the ionic concentrations at the initial and equilibrium time points, respectively; *Q*_*e*_ (mg/g) is the number of ions adsorbed on the adsorbing medium at equilibrium; *Q*_*max*_ (mg/g) is the maximum adsorption capacity of the adsorbent; and *K*_*L*_ and *K*_*F*_ are the Langmuir and Freundlich adsorption constants, respectively.

### Adsorption kinetics of target ions on MXene/AgNW composite material

For the adsorption kinetics study, 5 mg of the MXene/AgNW composite material was treated with 100 mL of Cs^+^ or I^−^ solution at concentration (*C*_*o*_) of 100 ppm, pH 7, and 25 °C. At various time intervals, the adsorbent was separated from the solution and the concentration of Cs^+^ or I^−^ was determined. The adsorption capacity was investigated using the pseudo-first-order (PFO) and pseudo-second-order (PSO) kinetic equations over time, as represented in Eqs. (5) and (6), respectively.

5 $$\mathrm{ln}\left({\mathrm{Q}}_{\mathrm{e}}-{\mathrm{Q}}_{\mathrm{t}}\right)=\mathrm{ ln}{\mathrm{Q}}_{\mathrm{e}}- \frac{{\mathrm{k}}_{1}\mathrm{t}}{2.303}$$

6 $$\frac{t}{{Q}_{t}}= \frac{1}{{k}_{2}{Q}_{e}^{2}}+ \frac{t}{{Q}_{e}}$$

Here, *Q*_*e*_ and *Q*_*t*_ are the quantities of the target ions (mg/g) at equilibrium and at time = *t*, respectively, and *k*_*1*_ (min^−1^) and *k*_*2*_ (g mg^−1^ min^−1^) are the PFO and PSO adsorption rate constants, respectively.

### Treatment of radioactive sample using MXene/AgNW

The efficacy of the MXene/AgNW composite was assessed using a radioactive sample containing radioactive iodine (^131^I). To measure the effectiveness of the adsorbent, Radio-thin-layer chromatography (TLC), a method using radiation detection and TLC, was employed. In total, 10 mg of MXene/AgNW composite was exposed to an aqueous solution of radioactive iodine (100 μCi/10 mL) under normal environmental conditions for 30 min. After the completion of the experiments, the adsorbent was separated using of centrifugation. The concentration of ^131^I^−^ within both the solution and adsorbent was measured using Radio-TLC. The TLC plates were developed using 0.9% saline solution as the eluent.

## Conclusion

The novel 1D/2D hybrid MXene/AgNW composite material, synthesized using 1D AgNW and 2D MXene demonstrated excellent efficiency for the simultaneous removal of Cs^+^ and I^−^ from the aqueous media. The 2D MXene possesses negatively charged surface functional groups and a porous surface structure, which results in a high removal efficiency for Cs^+^ even under diverse conditions and in the presence of competitive ions. The *Q*_*max*_ of the MXene/AgNW composite for Cs^+^ was 26.22 mg/g. The presence of AgNW did not affect the Cs^+^ adsorption capacity of the material, indicating that the MXene component was responsible for Cs^+^ adsorption. Additionally, the MXene/AgNW composite demonstrated remarkable removal efficiency for I^−^, with a *Q*_*max*_ 84.70 mg/g under various conditions, which was notably better than that of other reported materials. The adsorption kinetics of the Cs^+^ and I^−^ followed the PSO kinetics, indicating the importance of chemical adsorption mechanisms. Furthermore, the MXene/AgNW composite material was highly effective in removing radioactive iodine (^131^I) from the aqueous media. The results of our study suggest that the MXene/AgNW composite is a promising adsorbent and its adsorption capacities may be capable of removing ^137^Cs (17.0 μg/L) and ^129^I (3.07 ng/L) found at the Fukushima site.

### Supplementary Information

Supplementary Information.

## Data Availability

All data generated or analyzed during this study are included in this published article and its supplementary information files.
