# Supplementary material for: MXene/AgNW composite material for selective and efficient removal of radioactive cesium and iodine from water
Source: Sci Rep. 2023 Nov 11;13:19696. doi: 10.1038/s41598-023-47075-y (PMC10640589; doi:10.1038/s41598-023-47075-y)
Supplement: Supplementary file 1 — Supplementary Information. [file 41598_2023_47075_MOESM1_ESM.docx]

**Selective and efficient removal of radioactive cesium and iodine using MXene/AgNW composite material from contaminated water**

Sajid Mushtaq^a,b,*^, Syed M. Husnain^c^, Syed Asad Raza Kazmi^d^, Yawar Abbas^e^, Jongho Jeon^f^, Jung Young Kim^b^, Faisal Shahzad^a,*^

^a^Department of Nuclear Engineering, Pakistan Institute of Engineering and Applied Sciences, P. O. Nilore, Islamabad 45650, Pakistan

^b^Division of RI-Applied Research, Korea Institute of Radiological & Medical Sciences (KIRAMS), Seoul 01812, Korea

^c^Chemistry Division, Directorate of Science, Pakistan Institute of Nuclear Science and Technology (PINSTECH), Islamabad 45650, Pakistan

^d^Department of Metallurgy and Materials Engineering, Pakistan Institute of Engineering and Applied Sciences, P. O. Nilore, Islamabad 45650, Pakistan

^e^Department of Physics, Khalifa University, Abu Dhabi 127788, United Arab Emirates

^f^Department of Chemistry, Kyungpook National University, Daegu 80, Republic of Korea

*****Corresponding Authors

**E-mail**: sajidmushtaq@pieas.edu.pk; faisal@pieas.edu.pk


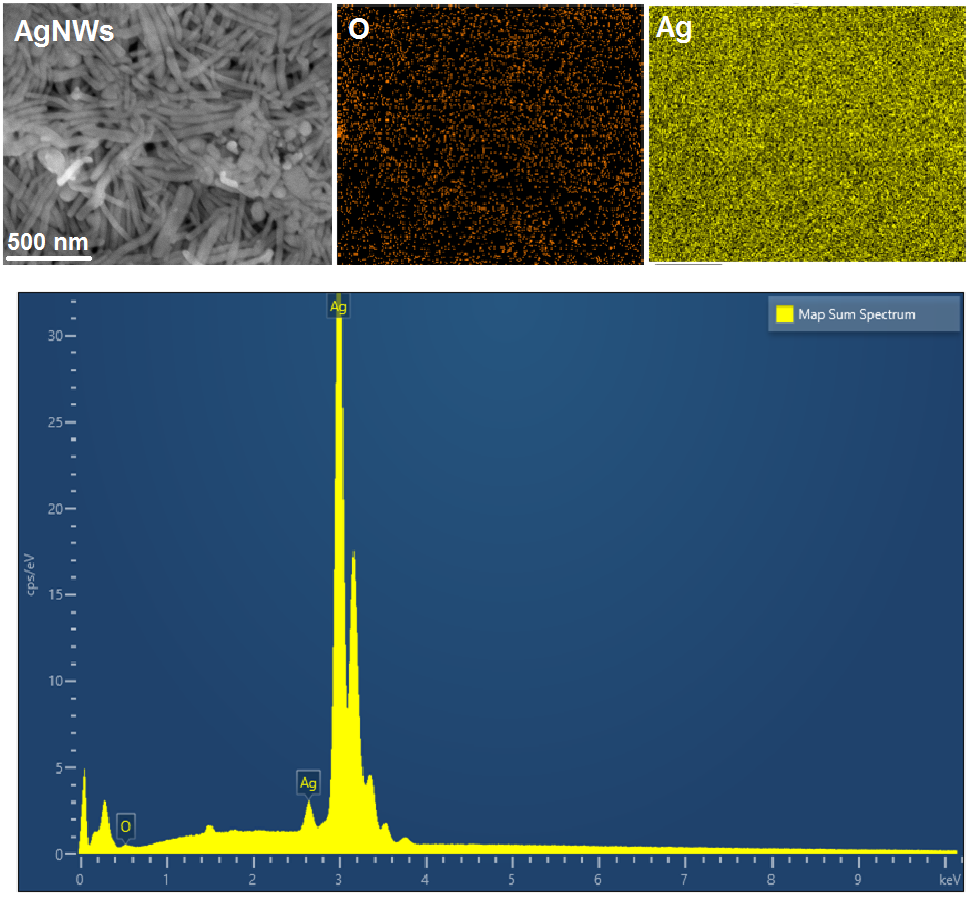


**Figure S1.** SEM image of Silver nanowires (AgNW), elemental mapping and EDS spectrum showing the presence of Ag, and O


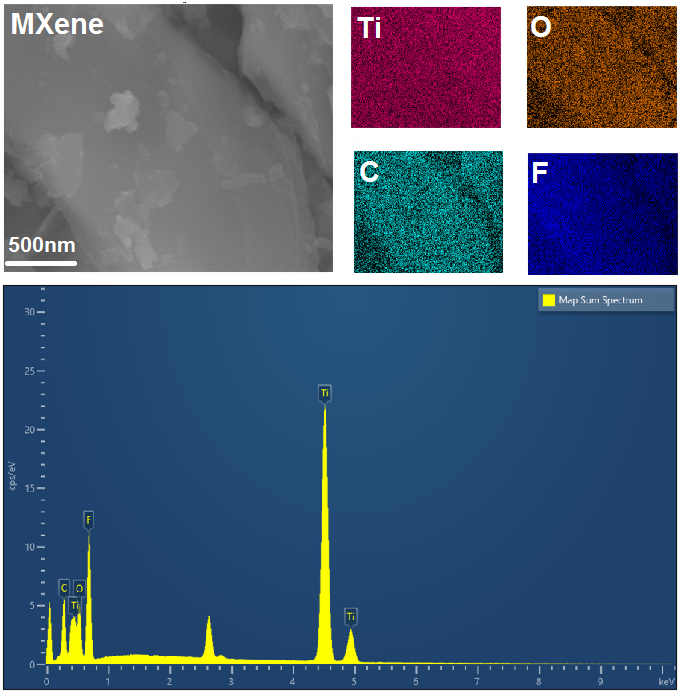


**Figure S2.** SEM image of MXene, elemental mapping and EDS spectrum showing the presence of Ti, O, C, and F


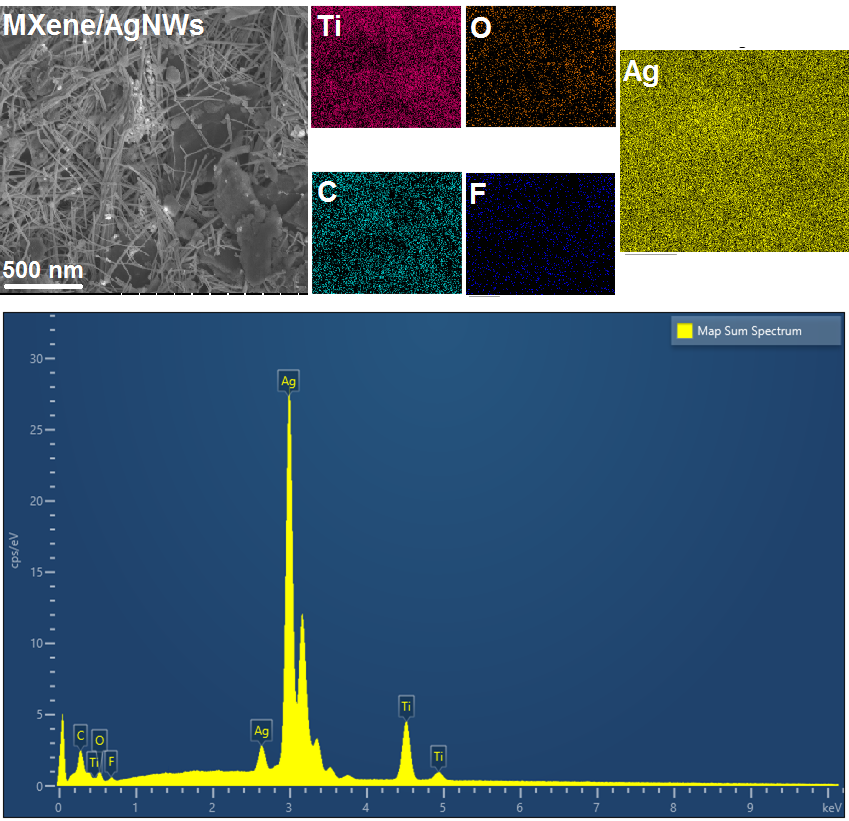


**Figure S3.** SEM image of MXene/AgNW, elemental mapping and EDS spectrum showing the presence of Ti, O, C, F, and Ag


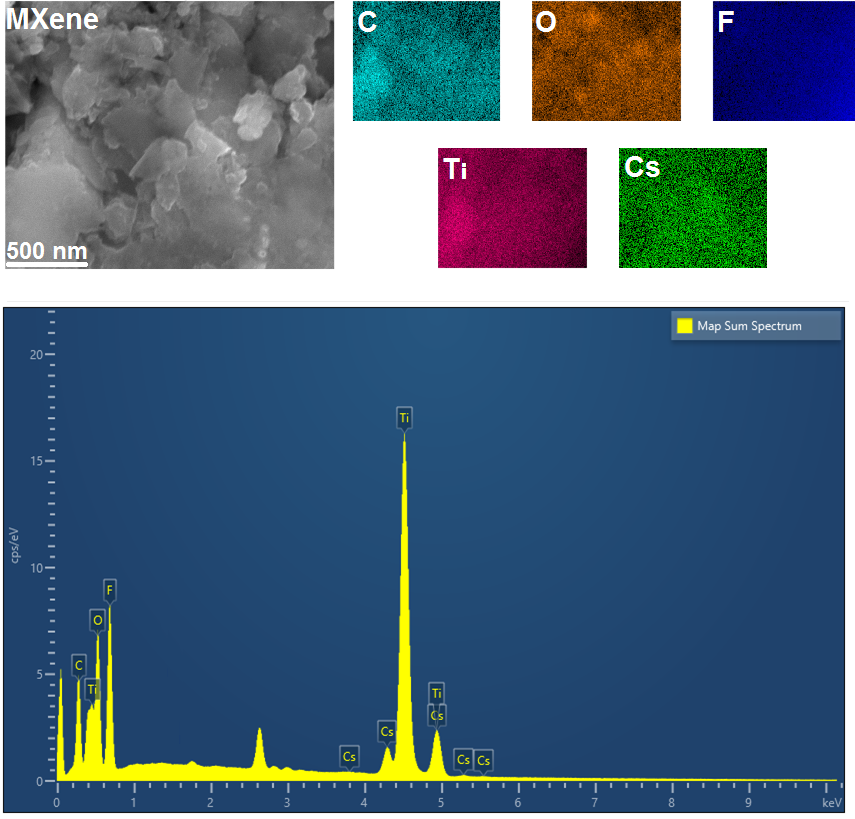


**Figure S4.** SEM image of MXene after the removal of Cs^+^ ions, elemental mapping and EDS spectrum showing the presence of C, O, F, Ti, and Cs

**
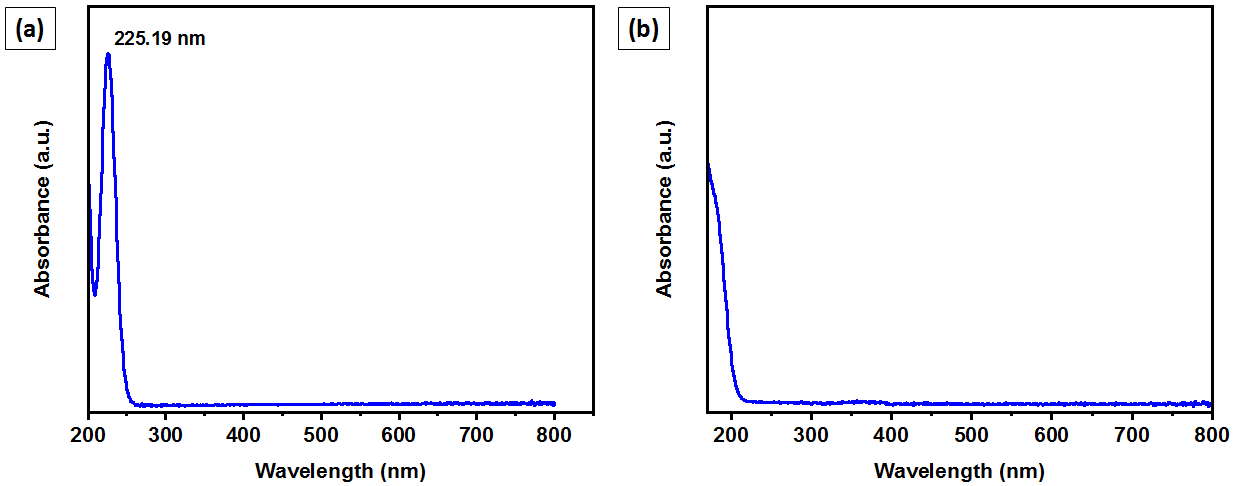
**

**Figure S5.** U-visible spectra of NaI solution (a) before treatment with MXene/AgNW, (b) after treatment with MXene/AgNW


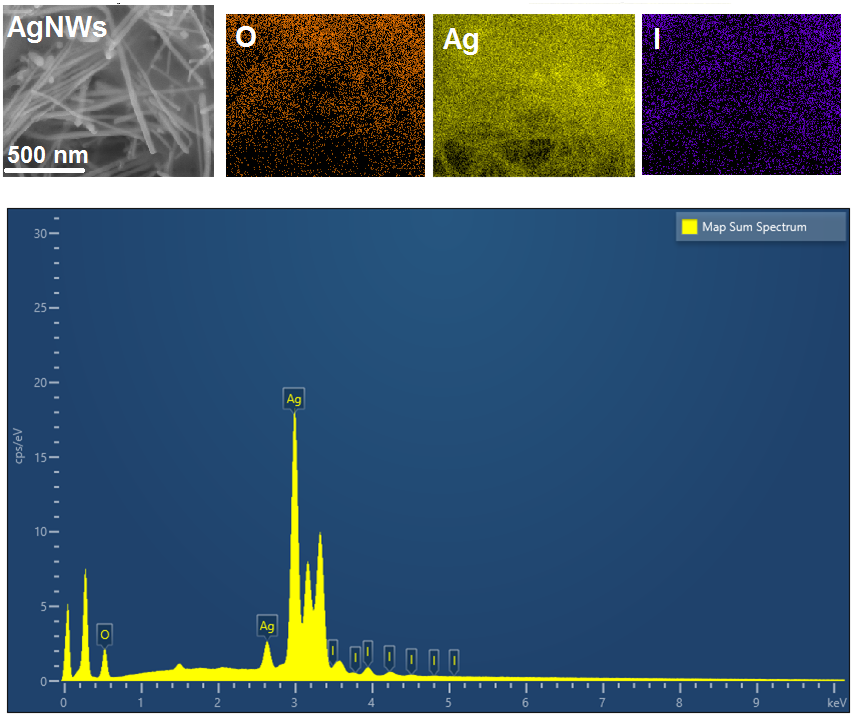


**Figure S6.** SEM image of AgNW after the removal of iodide ion, elemental mapping and EDS spectrum showing the presence of Ag, O, and I


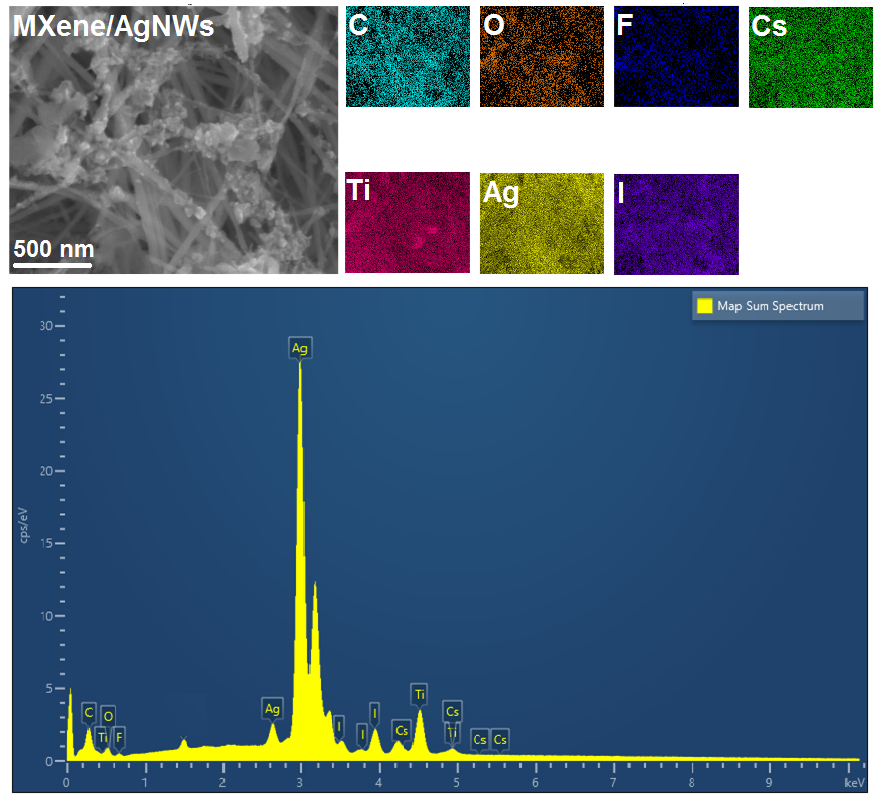


**Figure S7.** SEM image of MXene/AgNW composite after removal of cesium and iodide ions, elemental mapping and EDS spectrum showing the presence of C, O, F, Cs, Ti, Ag, and I

**Table S1.** Quantitative analysis of the adsorption efficacy of iodide and cesium ions by the MXene/AgNW composite vis-à-vis alternative adsorbents

| **Adsorbent** | **Q_max_ (mg/g) cesium ions** | **Q_max_ (mg/g) iodide ions** | **Specific conditions** | **Ref.** |
| --- | --- | --- | --- | --- |
| Ag-Ag_2_O coated carbon sphere | N.A. | 374. 91 | pH: 1.5˗2.1  Time: 1h | 1 |
| Ag_2_O / sodium niobate nanofibers | N.A. | 193.04 | pH: 7.0˗10.0  Time: 2h | 2 |
| MIL-101(Cr)-SO_3_Ag | N.A. | 244.2 | pH: 7.0˗10.0  Time: 24h | 3 |
| Ag loaded Cu_2_O | N.A. | 25.40 | pH: 7.0˗10.0  Time: 2h | 4 |
| Ag-doped carbon aerogels | N.A. | 251.47 | pH: 7.0˗10.0  Time: 7 days | 5 |
| Carbon aerogels doped with Ag(I) | N.A. | 17.78 | pH: 7.0  Time: 7 days | 6 |
| Silver-Impregnated Activated Carbon | N.A. | 340.60 | pH: 4.5  Time: 14 days | 7 |
| Calcium alginate–silver chloride composite | N.A. | 133.0 | pH: 6.0  Time: 48h | 8 |
| Cuprite (Cu_2_O) | N.A. | 0.114 | pH: 8.7  Time: 12h | 9 |
| Montmorillonite–iron oxide composite (MIOC) | 52.6 | N.A. | pH: 7.0˗10.0  Time: 3 h | 10 |
| layered sulfide material K_2_*_x_*Mn*_x_*Sn_3−_*_x_*S_6_ | 226.0 | N.A. | pH: 7.0˗10.0  Time: 12 h | 11 |
| Copper Hexacyanoferrate Nanoparticles on Mesoporous Silica | 286.1 | N.A. | pH: 7.0  Time: 242 h | 12 |
| Sodium titanosilicate-polyacrylonitrile (STS-PAN) composite | 22.5 | N.A. | pH: 6.0  Time: 2 h | 13 |
| Copper(II) ferrocyanide on mesoporous silica (FC-Cu-EDA-SAMMS | 17.1 | N.A. | pH: 7.0  Time: 1 h | 14 |
| Prussian-blue-modified magnetite (PB-Fe_3_O_4_) | 16.25 | N.A. | pH: 5.5  Time: 1 h | 15 |
| Multiwall carbon nanotubes (MWCNTs) | 12.77 | N.A. | pH: 7.5  Time: 1.5 h | 16 |
| Nickel hexacyanoferrate (NiHCF) | 4.95 | N.A. | pH: 7.0  Time: 2 h | 17 |
| Ammonium molybdophosphate (AMP)/ polyvinyl alcohol (PVA)/ sodium alginate (SA) composite hydrogel | 71.28 | N.A. | pH: 7.0  Time: 12 h | 18 |
| NiSiO@NiAlFe layered double hydroxides (LDHs) hollow spheres | 61.5 | N.A. | pH: 10.0  Time: 20 min | 19 |
| Prussian blue functionalized layered double hydroxide (PB-LDH) | 32.36 | 85.47 | pH: 4.0˗10.0  Time: 3 days | 20 |
| MXene/AgNW composite | 26.22 | 84.70 | pH: 7.0-9.0  Time: 60 min | This study |

The MXene/AgNW composite demonstrates efficient removal of significant quantities of cesium and iodide ions at moderate pH levels within a short duration. The composite exhibits superior adsorption capabilities when compared to those reported in existing literature

**References**

1. Yu, F., Chen, Y., Wang, Y., Liu, C. & Ma, W. Enhanced removal of iodide from aqueous solution by ozonation and subsequent adsorption on Ag-Ag_2_O modified on Carbon Spheres. *Appl. Surf. Sci*. **427**, 753-762 (2018).
2. Mu, W., Yu, Q., Li, X., Wei, H. & Jian, Y. Niobate nanofibers for simultaneous adsorptive removal of radioactive strontium and iodine from aqueous solution. *J. Alloys Compd.* **693**, 550-557 (2017).
3. Zhao, X., Han, X., Li, Z., Huang, H., Liu, D. & Zhong, C. Enhanced removal of iodide from water induced by a metal-incorporated porous metal–organic framework. *Appl. Surf. Sci.* **351**, 760-764 (2015).
4. Mao, P., Liu, Y., Jiao, Y., Chen, S. & Yang, Y. Enhanced uptake of iodide on Ag@ Cu_2_O nanoparticles. *Chemosphere*. **164**, 396-403 (2016).
5. Sánchez-Polo, M., Rivera-Utrilla, J., Salhi, E. & von Gunten, U. Ag-doped carbon aerogels for removing halide ions in water treatment. *Water Res*. **41**, 1031-1037 (2007).
6. Sánchez-Polo, M., Rivera-Utrilla, J., Méndez-Díaz, J. & López-Peñalver, J. Metal-doped carbon aerogels. New materials for water treatments. *Ind. Eng. Chem. Res*. **47**, 6001-6005 (2018).
7. Hoskins, J. S., Karanfil, T. & Serkiz, S. M. Removal and sequestration of iodide using silver-impregnated activated carbon. *Environ. Sci. Technol*. **36**, 784-789 (2002).
8. Zhang, H. *et al*. Adsorption of iodide ions on a calcium alginate–silver chloride composite adsorbent. *Colloids Surf. A: Physicochem. Eng. Asp*. **386**, 166-171 (2011).
9. Lefèvre, G., Walcarius, A., Ehrhardt, J. J. & Bessière, J. Sorption of iodide on cuprite (Cu_2_O). Langmuir. **16**, 4519-4527 (2000).
10. Ararem, A., Bouras, O. & Bouzidi, A.  Batch and continuous fixed-bed column adsorption of Cs^+^ and Sr^2+^ onto montmorillonite–iron oxide composite: comparative and competitive study. J. Radioanal. Nucl. Chem. **298**, 537-545 (2013).
11. Manos, M. J. & Kanatzidis, M. G. Highly efficient and rapid Cs^+^ uptake by the layered metal sulfide K_2x_ Mn_x_ Sn_3−x_ S_6_ (KMS-1). J. Am. Chem. Soc. **131**, 6599-6607 (2009).
12. Turgis, R. *et al*. An original “click and bind” approach for immobilizing copper hexacyanoferrate nanoparticles on mesoporous silica. *Chem. Mater*. **25**, 4447-4453 (2013).
13. Saberi, R., Nilchi, A., Rasouli Garmarodi, S. & Zarghami, R. Adsorption characteristic of ^137^Cs from aqueous solution using PAN-based sodium titanosilicate composite. J. Radioanal. Nucl. Chem. **284**, 461-469 (2010).
14. Sangvanich, T. *et al*. Selective capture of cesium and thallium from natural waters and simulated wastes with copper ferrocyanide functionalized mesoporous silica. *J. Hazard. Mater*. **182**, 225-231(2010).
15. Sasaki, T. & Tanaka, S. Magnetic separation of cesium ion using Prussian blue modified magnetite. *Chem. Lett*. **41**, 32-34 (2012).
16. Yavari, R., Huang, Y. & Ahmadi, S. Adsorption of cesium (I) from aqueous solution using oxidized multiwall carbon nanotubes. *J. Radioanal. Nucl. Chem*. **287**, 393-401(2011).
17. Ding, D. *et al*. Adsorption of cesium from aqueous solution using agricultural residue–walnut shell: equilibrium, kinetic and thermodynamic modeling studies. *Water Res*. **47**, 2563-2571 (2013).
18. Chen, S. *et al*. Composite hydrogel particles encapsulated ammonium molybdophosphate for efficiently cesium selective removal and enrichment from wastewater. *J. Hazard. Mater*. **371** 694-704 (2019).
19. Hu, Y. Y. *et al*. Prediction and optimization of adsorption properties for Cs+ on NiSiO@ NiAlFe LDHs hollow spheres from aqueous solution: Kinetics, isotherms, and BBD model. *J. Hazard. Mater*. **401**, 123374 (2021).
20. Kim, J., Kang, J. & Um, W. Simultaneous removal of cesium and iodate using prussian blue functionalized CoCr layered double hydroxide (PB-LDH). *J. Environ. Chem. Eng*. **10**, 107477 (2022).
